# Composite Noise Reduction Method for Internal Leakage Acoustic Emission Signal of Safety Valve Based on IWTD-IVMD Algorithm

**DOI:** 10.3390/s25154684

**Published:** 2025-07-29

**Authors:** Shuxun Li, Xiaoqi Meng, Jianjun Hou, Kang Yuan, Xiaoya Wen

**Affiliations:** 1 School of Petrochemical Technology, Lanzhou University of Technology, Lanzhou 730050, China; zhds00180081@126.com (S.L.); hou19700018@126.com (J.H.); 17393127867@163.com (K.Y.); 13919303595@163.com (X.W.); 2 Machinery Industry Pump Special Valve Engineering Research Center, Lanzhou 730050, China

**Keywords:** safety valve, acoustic emission (AE), variational mode decomposition (VMD), wavelet threshold denoising

## Abstract

As the core device for protecting the safety of the pressure-bearing system, the spring full-open safety valve is prone to various forms of valve seat sealing surface damage after long-term opening and closing impact, corrosion, and medium erosion, which may lead to internal leakage. In view of the problems that the high-frequency acoustic emission signal of the internal leakage of the safety valve has, namely, a large number of energy-overlapping areas in the frequency domain, the overall signal presents broadband characteristics, large noise content, and no obvious time–frequency characteristics. A composite denoising method, IWTD, improved wavelet threshold function with dual adjustable factors, and the improved VMD algorithm is proposed. In view of the problem that the optimal values of the dual adjustment factors a and b of the function are difficult to determine manually, an improved dung beetle optimization algorithm is proposed, with the maximum Pearson coefficient as the optimization target; the optimization is performed within the value range of the dual adjustable factors a and b, so as to obtain the optimal value. In view of the problem that the key parameters *K* and *α* in VMD decomposition are difficult to determine manually, the maximum Pearson coefficient is taken as the optimization target, and the improved dung beetle algorithm is used to optimize within the value range of *K* and *α*, so as to obtain the IVMD algorithm. Based on the IVMD algorithm, the characteristic decomposition of the internal leakage acoustic emission signal occurs after the denoising of the IWTD function is performed to further improve the denoising effect. The results show that the Pearson coefficients of all types of internal leakage acoustic emission signals after IWTD-IVMD composite noise reduction are greater than 0.9, which is much higher than traditional noise reduction methods such as soft and hard threshold functions. Therefore, the IWTD-IVMD composite noise reduction method can extract more main features out of the measured spring full-open safety valve internal leakage acoustic emission signals, and has a good noise reduction effect. Feature recognition after noise reduction can provide a good evaluation for the safe operation of the safety valve.

## 1. Introduction

As the core safety component of the industrial pipeline system, the reliable operation of safety valves is crucial to the overpressure protection and stability maintenance of pressure systems. However, internal leakage caused by seal failure may lead to medium leakage, equipment damage and even personal safety accidents. Therefore, the accurate detection of safety valve internal leakage has significant engineering value and safety significance. Acoustic emission (AE) detection technology, with its non-destructive characteristics, provides an effective method for valve internal leakage detection by capturing elastic wave signals caused by the turbulent vibration and micro-deformation of components when fluid flows through sealing defects. After the signal is collected by the sensor is amplified, filtered, and digitally processed, the internal leakage state can be identified through characteristic parameter analysis.

For the engineering application scenario of the spring full-lift safety valve, the quality of its detection signal is subject to dual challenges: on the one hand, the acoustic emission signal caused by internal leakage is essentially a low signal-to-noise ratio vibration signal with limited inherent resolution; on the other hand, complex industrial environmental noise, such as compressor vibration and pipeline mechanical noise, seriously pollutes the original signal, resulting in significant errors in feature parameter extraction, which in turn affects further research on valve internal leakage. Traditional noise reduction methods have problems of mode aliasing or feature loss when processing non-stationary, multi-component coupled signals, making it difficult to meet the needs of high-precision detection.

Wavelet noise reduction technology achieves effective separation of signal and noise through multi-resolution time–frequency analysis, while the variational mode decomposition (VMD) algorithm can adaptively decompose complex signals into a finite number of intrinsic mode functions. The combination of the two can suppress environmental noise interference while retaining the time–frequency characteristics of the internal leakage signal. This paper proposes a joint noise reduction method based on wavelet noise reduction and VMD optimization. By constructing a multi-scale feature fusion model, it solves the problem of feature extraction of the internal leakage signal of the spring full-open safety valve in a strong noise environment, and it provides a highly reliable signal preprocessing technology for valve internal leakage detection. It has important theoretical significance and engineering application value for improving the safety monitoring level of industrial pressure systems.

Digital filters use the randomness of signals, treating the signal and its noise as random signals, and use statistical characteristics to estimate the signal [1,2,3]. Wang et al. [4] used Kalman filters to study the denoising of helicopter main rotor and tail rotor aerodynamic noise, and obtained the main frequency band range of the noise of the two. Hesar et al. [5] used Bayesian filters to denoise electrocardiogram signals. The signal characteristics after denoising are prominent, which helps medical personnel diagnose characteristics. Although digital filtering has high reliability, it cannot adapt to non-stationary processes.

Wavelet transform can decompose the signal into several wavelet coefficients. The signal reconstructed by wavelet coefficients retains the local characteristic information of the original signal. The relevant domestic and foreign literature shows that wavelet threshold denoising shows good local characteristics in both time domain and frequency domain, can be used for multi-resolution analysis, and is suitable for the analysis and denoising of various types of signals [6,7,8,9]. Commonly used wavelet threshold denoising methods include hard threshold function denoising and soft threshold function denoising. Using hard threshold function to perform wavelet threshold denoising and reconstruct the signal leads to the occurrence of the signal oscillation Gibbs effect, resulting in the discontinuity of the reconstructed signal. The signal reconstructed after denoising using the soft threshold function has a constant deviation, resulting in the loss of the local feature information of the signal [10,11,12,13]. In recent years, scholars at home and abroad have proposed a variety of improved wavelet threshold functions [14,15] to address the defects of soft and hard threshold functions. For example, Sun et al. [16] proposed a sinusoidal improved threshold function to denoise the ultrasonic detection signal of EMU wheels. After denoising, the waveform characteristics of the wheel defect signal are retained, which is conducive to defect identification. Although the above studies proposed improved wavelet threshold functions with better denoising effects than traditional soft and hard threshold functions, most of them are single adjustment factor-improved threshold functions within or outside the absolute value range of the threshold, and the other range still retains the characteristics of the soft threshold or hard threshold function. In addition, the values of the adjustable factors of the improved threshold function in the existing literature all adopt the exhaustive adjustment method, and there is no case of using optimization algorithms to optimize the values of the adjustable factors.

The modal decomposition denoising method realizes feature extraction by the modal decomposition of the signal, thereby improving the signal-to-noise ratio of the signal. The empirical mode decomposition (EMD) method is a typical modal decomposition denoising method [17], which has been widely used in the field of signal denoising. EMD and its improved methods can decompose and reconstruct signals in specific modes and improve the signal-to-noise ratio of the signal. However, EMD is constrained by problems such as endpoint effects and modal aliasing, which affect its feature extraction and denoising effects [18]. In view of the shortcomings of the EMD method, many improved methods have been proposed, including the following: ensemble empirical mode decomposition (EEMD), local mean decomposition (LMD), complementary ensemble empirical mode decomposition (CEEMD), complete ensemble empirical mode decomposition with adaptive noise (CEEMDAN), etc. [19,20,21,22,23]. Although these algorithms make up for the shortcomings of EMD to a certain extent, they are often used as a preprocessing method due to their weak performance in noise suppression. The variational mode decomposition (VMD) algorithm is a completely non-recursive signal decomposition method proposed by Dragomiretskiy et al. [24]. The VMD algorithm can non-iteratively decompose the input signal and adaptively select the frequency band containing the most diagnostic information. It can also overcome modal aliasing and effectively suppress noise. Since its proposal, it has attracted widespread interest from researchers. For example, Tao et al. [25] used the VMD algorithm to study the feature extraction of urban water supply pipeline vibration signals, thereby achieving a preliminary judgment on the health status of the pipeline. Patil et al. [26] used the VMD algorithm to extract features from the sleeve vibration signal and selected the IMF component using the maximum peak criterion. However, in the VMD decomposition process, the key parameters *K* and *α* need to be continuously adjusted to adapt to the signal, which is time-consuming and labor-intensive.

As a classic benchmark in the field of wavelet denoising, the soft and hard threshold methods are simple in principle and widely used. Therefore, the original article uses them as the basic comparison object to measure the composite denoising effect. However, this type of method was proposed earlier, and it is difficult to fully reflect the progress of current signal processing technology. In recent years, improved modal decomposition methods (such as CEEMDAN) and variational mode decomposition (VMD) technology have provided better ideas for non-stationary acoustic emission signal processing. To this end, this paper adds a systematic comparison of three types of methods (traditional threshold, improved modal decomposition method, and standard VMD) to fully verify the advancement and feasibility of IWTD-IVMD.

Most of the above studies use wavelet threshold denoising or eigen decomposition to denoise the signal, but the denoising effect is not ideal, and no denoising study of high-frequency acoustic emission signals of valve internal leakage is included. In order to provide a relatively pure input signal for further research on the internal leakage of the spring full-open safety valve, it is necessary to reduce the noise and extract the features of the acoustic emission signal of the internal leakage of the safety valve. Therefore, two noise reduction methods, namely the combined and improved wavelet threshold function IWTD noise reduction and the improved VMD decomposition, are proposed to perform composite noise reduction on the acoustic emission signal of the internal leakage of the safety valve. In addition, in view of the problems that the adjustable parameters of the wavelet threshold function are difficult to select and the decomposition modulus *K* and the penalty factor *α* in the VMD decomposition are difficult to determine artificially, an improved method for optimizing the parameters of the optimization algorithm is proposed.

## 2. Constructing the Improved Wavelet Threshold Denoising IWTD Function

### 2.1. Wavelet Basis, Decomposition Level Selection, and Threshold Calculation Method

#### 2.1.1. Selection of Wavelet Basis

In wavelet analysis, there are many types of mother wavelets that can be used for wavelet analysis. Different mother wavelets will produce different results when used to analyze the same signal. In order to achieve a good wavelet threshold denoising effect, the mother wavelet should have characteristics such as orthogonality, compact support, symmetry, and vanishing moment. However, there are usually multiple mother wavelets with the same properties. Commonly used wavelet basis functions include Haar wavelet, Daubechies wavelet series, biorthogonal wavelet series, Coiflet wavelet series, Symlets wavelet series, Morlet wavelet, Mexican Hat wavelet, etc. According to the relevant domestic and foreign literature, the Daubechies wavelet series and the Symlets wavelet series both have good characteristics such as orthogonality, compact support, symmetry, and vanishing moment. Among them, the Daubechies wavelet is suitable for the study of wavelet threshold denoising that contains both low-frequency noise and high-frequency mechanical fault signals [27]. Considering the data acquisition environment of acoustic emission signals of various types of internal leakage of spring full-lift safety valves, it contains both high-frequency internal leakage signals and mechanical noise such as compressor vibration noise and pipeline vibration noise. Therefore, the Daubechies wavelet is selected as the wavelet basis function for the wavelet threshold denoising of acoustic emission signals of the spring full-open safety valve with no internal leakage and various types of internal leakage.

#### 2.1.2. Quantitative Selection of Decomposition Levels

The calculation method of the number of decomposition layers is constructed by using the approximate coefficient *C* and detail coefficient *D* after discrete wavelet transform decomposition. For the noise variance estimation of the noisy spring full-open safety valve internal leakage signal, the modulus of the *n*-th layer wavelet detail coefficient of the acoustic emission signal is arranged in descending order: {|*D_n_*_1_| > |*D_n_*_2_| > |*D_n_*_3_| > … > |*D_nk_*|}, and the wavelet coefficient *D_n_*_(*k*/2)_ of the middle value is taken, then the standard deviation of each layer of noise estimation is as follows:

(1) $$ED_{sn(n,k)}=\frac{D_{n\frac{k}{2}}}{0.6745}$$

Considering that the wavelet coefficient value of the noise contained in the internal leakage acoustic emission signal of various types of spring full-open safety valves at the high level is much smaller than the signal wavelet coefficient, the wavelet coefficient of the nth layer is distinguished by threshold value:

(2) $$D_{\left(n,k\right)}^{\prime }=\left\{\begin{array}{cc} D_{s(n,k)} & \left|D_{s(n,k)}-{\bar{D}}_{sn(n,k)}\right|\geq 2ED_{sn(n,k)}\\ D_{s(n,k)}-{\bar{D}}_{sn(n,k)} & \left|D_{s(n,k)}-{\bar{D}}_{sn(n,k)}\right|\leq 2ED_{sn(n,k)} \end{array}\right.$$

where *D_s_*_(*n*,*k*)_ is the *n*th and *k*th wavelet detail coefficients of the internal leakage acoustic emission signal of the noisy spring full-open safety valve, and ${\bar{D}}_{sn(n,k)}$ is the mean value of the nth wavelet detail coefficients of the noise contained.

The wavelet coefficient layering coefficient *Nd* is as follows:

(3) $$Nd=\frac{{\left\|C_{n}\right\|}^{2}+{\left\|D_{\left(n,k\right)}^{\prime }\right\|}^{2}}{{\left\|C_{n}\right\|}^{2}+{\left\|D_{\left(n,k\right)}\right\|}^{2}}$$

The threshold *λ_η_* of the stratification coefficient is set to 0.9196 [28]. When *Nd* > *λ_η_*, it can be considered that the low-frequency signal contains noise wavelet coefficients and the decomposition continues. When *Nd* < *λ_η_*, the noise wavelet coefficient becomes larger and it can be considered that the signal has been decomposed to the low frequency of the noise and the decomposition stops.

#### 2.1.3. Wavelet Threshold Selection and Improvement

Most existing threshold calculation methods use a fixed threshold for each layer of wavelet coefficients. However, the coefficients of each layer of the signal are different under wavelet decomposition, so it is not reasonable to use a fixed threshold calculation method for each layer [29]. The modulus of the *n*th layer of the wavelet coefficients of the spring full-open safety valve internal leakage acoustic emission signal is sorted from large to small, so that {|*D_n_*_1_| > |*D_n_*_2_| > |*D_n_*_3_| > … > |*D_nk_*|}. The mean of the first three largest wavelet coefficients is taken as the wavelet peak. At this time, the value of the mean peak-to-peak ratio is as follows:

(4) $$Apr_{n}=\frac{\left|D_{n1}\right|+\left|D_{n2}\right|+\left|D_{n3}\right|}{3\sum_{k=1}^{N_{n}}\left|D_{n,k}\right|}$$

When the *i*-th layer *Apr* is particularly small, the *i*-th layer can be completely filterable noise wavelet coefficients. At this time, the *i*-th layer *Apr_i_* is the noise coefficient cutoff value, and the modified threshold expression is as follows:

(5) $$\lambda_{n}=L_{i}\frac{\sigma \sqrt{2\log_{2}N}}{\log_{2}\left(n+1\right)}$$

where, *σ* = *ED_sn_*_(*n*,*k*)_, *L_i_* is the mean peak-to-peak ratio correction factor, and *N* is the measured signal length:

(6) $$L_{i}=2\left|\frac{Apr_{j}-Apr_{i}}{Apr_{j}+Apr_{i}}\right|$$

### 2.2. Establishment of Improved Wavelet Threshold Denoising IWTD Function

Assume that the collected acoustic emission signals of various internal leakage types of spring full-open safety valves can be described as:

(7) $$x\left(n\right)=v\left(n\right)+t\left(n\right)$$

where, *v*(*n*) is the real information of the acoustic emission signal of each type of internal leakage of the spring full-open safety valve; *t*(*n*) is the noise information of the measured environment. Wavelet transform of *x*(*n*) can be decomposed into the following:

(8) $$\omega_{x}\left(j,k\right)=\omega_{v}\left(j,k\right)+\omega_{t}\left(j,k\right)$$

where, *j* = 0, 1, 2, …, *J*; *k* = 0, 1, 2, …, *N*. *J* is the maximum wavelet decomposition layer number; *N* is the scale of the internal leakage acoustic emission information. After wavelet processing, each internal leakage acoustic emission signal will form multiple wavelet coefficients, which contain important characteristic information. The threshold is used as the boundary to distinguish between noise and signal, retaining important information with large coefficients, removing noise with small coefficients, reorganizing the processed wavelet coefficients, and obtaining the denoised signal.

**Figure 1:**
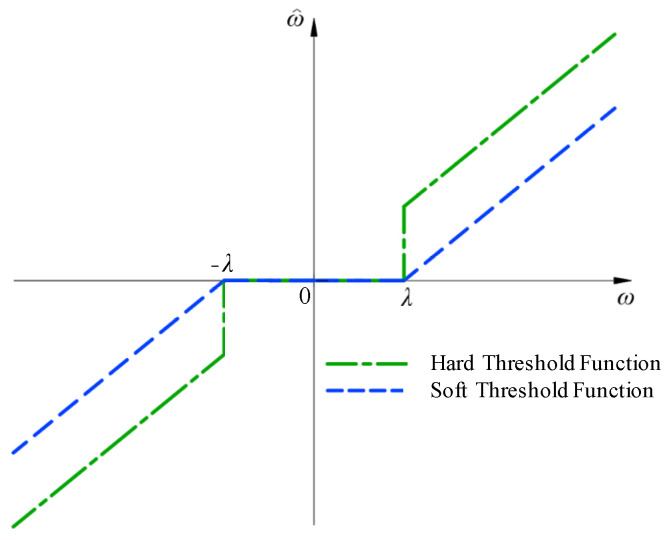
Hard threshold and soft threshold function diagram.

The traditional wavelet threshold function is the hard threshold function and soft threshold function proposed by Donoho [30], as shown in Figure 1, and their expressions are as follows:

(9) $${\hat{\omega }}_{h}=\left\{\begin{array}{cc} \omega , & \left|\omega \right|\geq \lambda \\ 0, & \left|\omega \right|<\lambda \end{array}\right.$$

(10) $${\hat{\omega }}_{s}=\left\{\begin{array}{cc} \mathrm{sign}(\omega )(\left|\omega \right|-\lambda ), & \left|\omega \right|\geq \lambda \\ 0, & \left|\omega \right|<\lambda \; \end{array}\right.$$

(11) $$\lambda =\hat{\sigma }\sqrt{2\ln N}$$

where, *N* is the signal length and $\hat{\sigma }$ is the estimated value of the noise standard deviation. For signals containing Gaussian noise, robust estimation $\hat{\sigma }$ can be performed by using the median absolute deviation (MAD) of the finest scale coefficients of wavelet decomposition:

(12) $$\hat{\sigma }=\frac{\mathrm{median}(|\omega_{1,k}|)}{0.6745}$$

where, *ω*_1_,*_k_* is the first layer wavelet coefficient. The universal threshold method can effectively suppress Gaussian noise while retaining the signal characteristics, and the calculation is simple, which is suitable for the broadband noise characteristics of the safety valve leakage acoustic emission signal.

As shown in Figure 1, the hard threshold function sets the points with absolute values less than the threshold to zero, while retaining the points with absolute values greater than the threshold. However, the hard threshold function has discontinuities at the positive and negative thresholds, and the reconstructed signal will produce oscillation and blurred Gibbs effects. The soft threshold function is continuous at the threshold, but it shrinks as a whole, and the reconstructed signal has a constant deviation from the original signal.

For the threshold function, when the function is continuous at the threshold and is not directly set to zero within [−*λ*, *λ*], it has a good noise reduction effect. On this basis, if the function approaches the hard threshold function, the deviation of the reconstructed signal can be effectively reduced, and a more accurate signal can be obtained. Therefore, an adjustable dual-parameter-improved wavelet threshold denoising function is proposed. By introducing an exponential function and adjusting parameters *a* and *b*, a dual-parameter adjustable wavelet threshold function with continuity, flexibility, and small constant deviation is proposed. When the coefficient of the wavelet decomposition is lower than the threshold, the proposed dual-parameter adjustable improved wavelet threshold function is not directly set to 0, which can obtain a better model noise reduction effect. The expression of the improved wavelet threshold denoising IWTD function is shown in Formula (13):

(13) $$\hat{\omega }=\left\{\begin{array}{c} \mathrm{sign}(\omega )\left[\left|\omega \right|+2\lambda \frac{\left(a-1\right)}{e^{(1-b){\left(\omega -\lambda \right)}^{2}}+1}\right],\;\left|\omega \right|\geq \lambda \\ \mathrm{sign}(\omega )a\left|\omega \right|e^{(1-b){\left(\omega -\lambda \right)}^{2}},\;\left|\omega \right|<\lambda \end{array}\right.$$

In the formula: *ω* is the wavelet coefficient of the original signal; *λ* is the threshold; $\hat{\omega }$ is the wavelet coefficient of the denoised signal; and *a* and *b* are adjustment factors.

Construct the function *f*(*ω*) = $\hat{\omega }$, verify the properties of the function from the following aspects, and prove the feasibility of the IWTD function based on mathematical derivation. The mathematical proof of the IWTD function is as follows:

(1) Continuity of IWTD function:

When $\omega \to \lambda^{+}$, we get Equation (14):

(14) $$\underset{\omega \to \lambda^{+}}{\lim}f(\omega )=\underset{\omega \to \lambda^{+}}{\lim}\mathrm{sign}(\lambda )\left[\lambda +2\lambda \frac{\left(a-1\right)}{1+e^{(1-b){\left(\lambda -\lambda \right)}^{2}}}\right]=\lambda a$$

When $\omega \to \lambda^{-}$, we get Formula (15):

(15) $$\underset{\omega \to \lambda^{-}}{\lim}f(\omega )=\underset{\omega \to \lambda^{-}}{\lim}\mathrm{sign}(\lambda )a\lambda e^{(1-b){\left(\lambda -\lambda \right)}^{2}}=a\lambda$$

From the above formula, we can know that: $\underset{\omega \to \lambda^{+}}{\lim}f(\omega )=\underset{\omega \to \lambda^{-}}{\lim}f(\omega )$, so the IWTD function is continuous at *ω* = *λ*.

Similarly, we can get: $\underset{\omega \to -\lambda^{+}}{\lim}f(\omega )=\underset{\omega \to -\lambda^{-}}{\lim}f(\omega )$, and the IWTD function is also continuous at *ω* = −*λ*. Therefore, *f*(*ω*) is continuous.

The continuity of the IWTD function can avoid the problems of glitches, spikes, oscillations, etc. in the traditional hard threshold function during signal reconstruction. Therefore, the above formula proves that the IWTD function is continuous in the definition domain, eliminating the degradation of signal quality caused by spikes, oscillations, etc.

(2) Asymptotic property of IWTD function:

When ω→+∞, we get Equation (16):

(16) $$\begin{array}{l} \underset{\omega \to +\infty }{\lim}\frac{f(\omega )}{\omega }=\underset{\omega \to +\infty }{\lim}\frac{\omega +2\lambda \frac{\left(a-1\right)}{e^{(1-b){\left(\omega -\lambda \right)}^{2}}+1}}{\omega }\\ \;=\underset{\omega \to +\infty }{\lim}1+2\lambda \frac{\left(a-1\right)}{\omega \left[1+e^{(1-b){\left(\omega -\lambda \right)}^{2}}\right]}=1 \end{array}$$

When ω→−∞, we get Formula (17):

(17) $$\begin{array}{l} \underset{\omega \to -\infty }{\lim}\frac{f(\omega )}{\omega }=\underset{\omega \to -\infty }{\lim}\frac{-\left[-\omega +2\lambda \frac{\left(a-1\right)}{e^{(1-b){\left(\omega -\lambda \right)}^{2}}+1}\right]}{\omega }\\ \;=\underset{\omega \to +\infty }{\lim}1-2\lambda \frac{\left(a-1\right)}{\omega \left[e^{(1-b){\left(\omega -\lambda \right)}^{2}}+1\right]}=1 \end{array}$$

From Equations (16) and (17), we can see that: The asymptote of $\hat{\omega }$ is $\hat{\omega }=\omega$. The asymptote of the IWTD function gradually approaches the hard threshold value when it tends to infinity, which well preserves the local characteristics of the signal and reduces the influence of uncontrollable noise.

(3) Bias of the IWTD function:

When ω→+∞, we get Equation (18):

(18) $$\begin{array}{l} \underset{\omega \to +\infty }{\lim}f(\omega )-\omega =\underset{\omega \to +\infty }{\lim}\omega +2\lambda \frac{\left(a-1\right)}{e^{(1-b){\left(\omega -\lambda \right)}^{2}}+1}-\omega \\ \;\;=\underset{\omega \to +\infty }{\lim}2\lambda \frac{\left(a-1\right)}{e^{(1-b){\left(\omega -\lambda \right)}^{2}}+1}=0 \end{array}$$

When ω→−∞, we get Formula (19):

(19) $$\begin{array}{l} \underset{\omega \to -\infty }{\lim}f(\omega )-\omega =\underset{\omega \to -\infty }{\lim}-\left[-\omega +2\lambda \frac{\left(a-1\right)}{e^{(1-b){\left(\omega -\lambda \right)}^{2}}+1}\right]-\omega \\ \;\;=\underset{\omega \to +\infty }{\lim}-2\lambda \frac{\left(a-1\right)}{e^{(1-b){\left(\omega -\lambda \right)}^{2}}+1}=0 \end{array}$$

From Equations (18) and (19), we can see that when it approaches infinity, there is no constant deviation between $\hat{\omega }$ and ω of the IWTD function, which overcomes the constant deviation problem of the soft threshold function and can effectively reduce the loss of signal information. The ultimate goal of wavelet decomposition is to make $\left\|\hat{\omega }-\omega \right\|$ as small as possible, and the IWTD function meets this condition.

**Figure 2:**
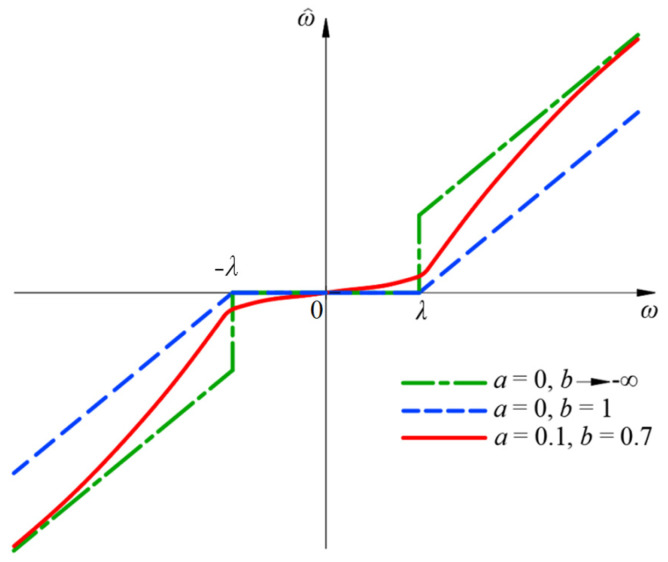
IWTD function diagram for different dual parameter values.

(4) Analysis of improved threshold function adjustment parameters:

Figure 2 is the IWTD function diagram under different values of adjustable factors *a* and *b*. When *a* = 0, *b* = 1, it approaches the soft threshold function. When *a* = 0, *b* tends to negative infinity, and it approaches the hard threshold function. Under other *a* and *b* values, the IWTD function has good continuity, asymptotes, and other advantages. Therefore, the analysis of Figure 2 shows that the corresponding IWTD function can be flexibly changed by changing the adjustment parameters *a* and *b* according to different signal forms to suit a variety of different occasions.

### 2.3. Optimization of Dual Adjustable Parameters of IWTD Function

#### 2.3.1. Dung Beetle Optimization Algorithm

Xue et al. [31] proposed the dung beetle optimization (DBO) algorithm in 2022. The algorithm has the advantages of high computational efficiency and is suitable for dealing with optimization problems with less than three parameters to be optimized.

Based on the rolling behavior of dung beetles, the position of dung beetles is updated according to Formula (20):

(20) $$\begin{array}{l} x_{i}\left(t+1\right)=x_{i}\left(t\right)+\alpha_{l}kx_{i}\left(t-1\right)+b_{l}\Delta x\\ \Delta x=\left|x_{i}\left(t\right)-x^{worst}\right| \end{array}$$

where, *t* represents the current iteration number; *x_i_*(*t*) is the position information of the *i*-th dung beetle in the *t*-th iteration; *k* is the deflection coefficient, with a value range of (0, 0.2]; *b_l_* is a constant, with a value range of (0, 1); *α*_l_ is a natural coefficient with a value of 1 or −1, 1 means no deviation, and −1 means deviation from the original direction; *x^worst^* represents the global worst dung beetle position; ∆*x* is used to simulate the change in light intensity.

Based on the dancing behavior of dung beetles, the position of dung beetles can be updated according to Formula (21):

(21) $$x_{i}\left(t+1\right)=x_{i}\left(t\right)+\tan\left(\theta_{s}\right)\left|x_{i}\left(t\right)-x_{i}\left(t-1\right)\right|$$

where, *θ_s_* is the deflection angle, and its value range is [0, π]. It should be noted that when *θ_s_* = 0, π/2 or π, the position of the dung beetle is not updated.

**Figure 3:**
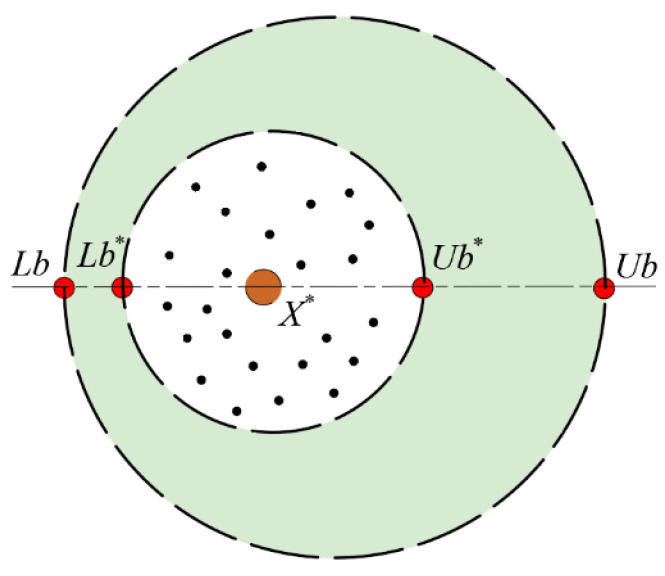
Boundary selection strategy.

Based on the reproduction behavior of dung beetles, the boundary selection strategy can be described by combining Figure 3 and Formula (22):

(22) $$\begin{array}{l} Lb^{*}=\max\left(X^{*}\times \left(1-R\right),Lb\right)\\ Ub^{*}=\min\left(X^{*}\times \left(1+R\right),Ub\right) \end{array}$$

where, X* represents the current local optimal position; Lb* and Ub* represent the lower and upper bounds of the egg-laying area, respectively; *R* = 1 − *t*/*T_max_*, *T_max_* represents the maximum number of iterations; *Lb* and *Ub* represent the lower and upper bounds of the optimization problem, respectively. It should be noted that the boundary range of the female dung beetle’s egg-laying area is dynamically adjusted with the number of iterations.

The position change of the egg can be described by the following formula (23):

(23) $$B_{i}\left(t+1\right)=X^{*}+b_{1}\left(B_{i}\left(t\right)-Lb^{*}\right)+b_{2}\left(B_{i}\left(t\right)-Ub^{*}\right)$$

where, *B_i_*(*t*) is the position information of the *i*-th egg ball at the *t*-th generation selection; *b*_1_ and *b*_2_ represent two independent random vectors of size 1 × *D_l_*, and *D_l_* represents the dimension of the optimization problem.

Based on the foraging behavior of dung beetles, the optimal foraging area of dung beetles and the position changes during foraging can be described by Equations (24) and (25):

(24) $$\begin{array}{l} Lb^{b}=\max\left(X^{b}\left(1-R\right),Lb\right)\\ Ub^{b}=\min\left(X^{b}\left(1+R\right),Ub\right) \end{array}$$

(25) $$x_{i}\left(t+1\right)=x_{i}\left(t\right)+C_{1}\left(x_{i}\left(t\right)-Lb^{b}\right)+C_{2}\left(x_{i}\left(t\right)-Ub^{b}\right)$$

where, *X^b^* represents the global optimal position; *Lb^b^* and *Ub^b^* are the lower and upper bounds of the optimal foraging area; *x_i_*(*t*) represents the position information of the *i*-th dung beetle in the *t*-th iteration; *C*_1_ represents a random number following a normal distribution; and *C*_2_ represents a random vector in the range of (0, 1).

Based on the stealing behavior of dung beetles, the position update of dung beetles is shown in Formula (26):

(26) $$x_{i}\left(t+1\right)=X^{b}+eg\left(\left|x_{i}\left(t\right)-X^{*}\right|+\left|x_{i}\left(t\right)-X^{b}\right|\right)$$

where, *x_i_*(*t*) is the position information of the *i*-th thief dung beetle at the *t*-th iteration, *g* is a random vector of size 1 × D that obeys the normal distribution, and *e* is a constant value.

#### 2.3.2. Establishment of the Improved Dung Beetle Optimization Algorithm

In solving optimization problems, the dung beetle optimization algorithm shows significant advantages such as high accuracy, fast convergence rate, and strong stability. However, there is still room for improvement in its global search capability. This section proposes three enhancement strategies for the dung beetle optimization algorithm, aiming to further accelerate the convergence speed of the algorithm and enhance its global search performance. The improved dung beetle optimization algorithm (IDBO) comprehensively enhances the optimization capability of the original DBO algorithm by introducing the greedy lens imaging inverse learning mechanism, integrating the optimal de-disturbance strategy of PID (proportional-integral-derivative) control, and adding curve adaptive factors.

Introducing the greedy lens imaging reverse learning mechanism in the population initialization stage: In theory, a high-quality optimization algorithm should have the characteristic that the final optimal solution is independent of the initial position, but for most random algorithms, this is not the case in practice. If the initial solution can be anchored in a dominant position in the population, the probability of the population converging to the optimal solution will be significantly improved, and it will also have a key impact on the convergence speed and solution accuracy of the algorithm [32]. Therefore, under the premise of the uniform random initialization of the population, a new population is generated by introducing the lens imaging reverse learning strategy, and high-quality individuals are selected from the combined population according to the fitness value to form a new population. This process helps to shorten the optimization time in the algorithm iteration process. The mathematical expression of the lens imaging reverse learning strategy is as follows:

(27) $$x_{j}(i+1)=\frac{{ub}_{j}+{lb}_{j}}{2}+\frac{{ub}_{j}+{lb}_{j}}{2k}-\frac{x_{j}\left(i\right)}{k}$$

where, *x_j_*(*i*) is the *i*-th individual in the *j*-dimension; *ub_j_* and *lb_j_* are the *j*-dimensional components of the upper and lower bounds of the decision variable, respectively; *k* is the scaling factor.

The PID control optimal de-disturbance strategy: During the algorithm iteration process, as the initial population individuals are continuously updated, the population diversity often loses a certain degree of diversity. In recent years, many researchers tend to adopt mutation-disturbance strategies to improve population diversity and obtain richer search information. For example, Guo et al. [32] introduced the position update mechanism of the follower in the sparrow search algorithm into the algorithm perturbation, and combined it with the Cauchy–Gaussian mutation strategy to help the algorithm escape from the local optimal solution; Pan et al. [33] introduced an adaptive Gaussian–Cauchy mixed mutation perturbation to enhance the coordination ability of the dung beetle algorithm between local development and global exploration. This paper designs an optimal de-disturbance method based on PID control to generate new individuals. PID control can promote population individuals to optimize in multiple directions, effectively increase population diversity, and improve algorithm search capabilities. In the field of control, the PID algorithm has excellent performance of quickly and stably outputting set values by virtue of the organic combination of proportional, integral, and differential control. In fitting regression problems, the algorithm usually optimizes based on the value of the fitness function [34]. Therefore, by regulating the optimal fitness function value through PID control and then fine-tuning the optimal individual, the algorithm can break through the limitation of local extreme values. The mathematical expression of PID control is as follows:

(28) $$u(k)=K_{p}err(k)+\frac{K_{p}T}{T_{i}}\sum_{n=0}^{k}err(n)+\frac{K_{p}T_{d}}{T}(err(k)-err(k-1))$$

where, *K_p_* is a proportional constant, usually *K_p_* = 0.4; *K_i_* = (*K_p_T*)/*T_i_* is an integral constant; *K_d_* = (*K_p_T_d_*)/*T* is a differential constant.

Curve adaptive weight factor: In order to optimize the coordination between global search and local exploration, and improve the algorithm’s iterative optimization ability and late convergence efficiency, this paper introduces a curve adaptive weight in the rolling dung beetle position update, Formula (30). This weight factor is designed based on the cosine function, and its change trend shows a cosine curve-like characteristic as the number of iterations increase. In the early stage of the iteration, the weight decreases at a relatively slow rate, allowing the algorithm to retain sufficient time to conduct a global search and reduce the risk of missing the global optimal solution by expanding the search range; in the later stage of the iteration, the weight decays rapidly, prompting the algorithm to quickly focus the search on the local optimal area and accelerate the convergence process. This mechanism can effectively enhance the global search performance of the algorithm and optimize the local optimization ability in the later stage. Its mathematical expression is as follows:

(29) $$w_{1}=w_{\min}+(w_{\max}-w_{\min})\times \frac{1+\cos(\pi \times {\left(\frac{t}{T_{\max}}\right)}^{2})}{2}$$

where, *t* is the current iteration number; *T_max_* is the maximum iteration number; *w_max_* and *w_min_* are the maximum and minimum values of the factor, respectively. In Formula (26), the adaptive weight of the curve is added and modified as follows:

(30) $$x_{i}(t+1)=w_{1}x_{i}(t)+\tan\theta_{s}|(1-w_{1})x_{i}(t)-x_{t}(t-1)|$$

#### 2.3.3. Selection of the Appropriate Function of the Improved Dung Beetle Optimization Algorithm

Since there are various mechanical noises in the actual measurement environment of the spring full-open safety valve, such as compressor vibration noise, pipeline vibration noise, and pipe system throttling element vibration noise, it is impossible to obtain a pure internal leakage acoustic emission signal that is completely free of noise. The commonly used signal-to-noise ratio combined with the root mean square error noise reduction effect evaluation method requires the pure signal to be known in advance, which is not applicable to this research background. The Pearson coefficient can well reflect the correlation between the main features of the denoised signal and the original signal, and the calculation amount is small. In recent years, it has been widely used as an evaluation indicator for signal processing in noisy environments. The Pearson coefficient is shown in Formula (31):

(31) $$R=\frac{\sum \left[x^{\prime }(t)-\bar{x^{\prime }(t)}\right]\left[x(t)-\bar{x(t)}\right]}{\sqrt{\sum {\left[x^{\prime }(i)-\bar{x^{\prime }(i)}\right]}^{2}}\sqrt{\sum {\left[x(t)-\bar{x(t)}\right]}^{2}}}$$

where, *x*(*t*) is the original signal; *x*′(*t*) is the signal after noise reduction.

The Pearson coefficient is between 0 and 1. When the Pearson coefficient is closer to 1, it means that the signal after noise reduction can better reflect the main characteristics of the original signal and the noise reduction effect is better. Therefore, the maximum Pearson coefficient is taken as the optimization target and the optimal function of the improved dung beetle optimization algorithm to obtain the optimal values of the adjustable parameters *a* and *b* that are suitable for the noise reduction of the acoustic emission signal of the spring full-open safety valve without internal leakage and various internal leakage types.

Although the Pearson coefficient can effectively measure the consistency of the main features of the denoised signal and the original signal, considering that the original signal itself contains noise, relying solely on this indicator may affect the objectivity of the evaluation. For this reason, an auxiliary evaluation system is introduced: the signal-to-noise ratio (SNR) that can quantify the noise suppression effect is calculated as follows:

(32) $$\mathrm{SNR}=10\log_{10}\left(\frac{\|v(n){\|}^{2}}{\|x^{\prime }(n)-v(n){\|}^{2}}\right)$$

where, *v*(*n*) is the pure signal.

Root mean square error (RMSE) evaluates the closeness of the denoised signal to the real signal; spectral flatness distinguishes broadband noise from narrowband acoustic emission characteristics. In the optimization process, the Pearson coefficient is still used as the main objective function because it is efficient in calculation and does not require a prior pure signal, but the final performance is ensured by multi-index cross-validation to ensure the denoising effect.

#### 2.3.4. Optimization of the Dual Adjustable Parameters of the Improved Wavelet Threshold Function

**Figure 4:**
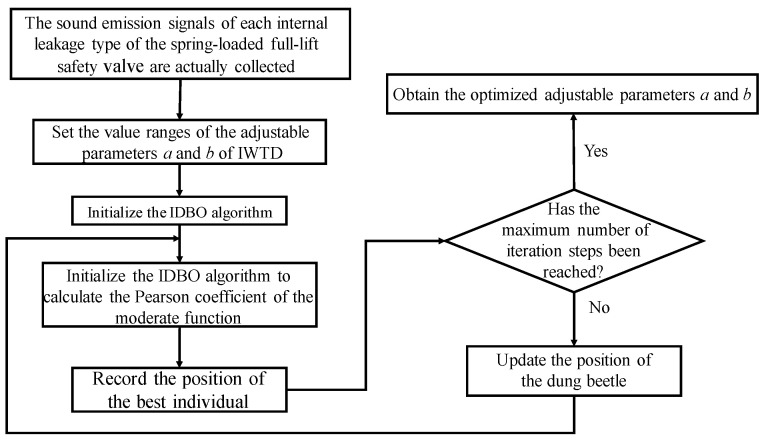
Flowchart of double parameter optimization for improved wavelet threshold function.

The optimization process of the dual adjustable parameters of the IWTD function is shown in Figure 4. The measured acoustic emission signal of the spring full-open safety valve is used as input, the adjustable parameter value range is set, and the IDBO optimization algorithm is initialized. The Pearson coefficient is used as the moderate function of the IDBO optimization algorithm, the best individual position in the iterative process is recorded, and the optimal values of the adjustable parameters a and b of the IWTD function are obtained.

## 3. Construct IVMD Algorithm to Extract Internal Leakage Signal Features

### 3.1. Principle of VMD Algorithm

VMD is an adaptive signal processing method. The key lies in the construction and solution of variational problems. The model of the unconstrained variational problem is as follows:

(33) $$\begin{array}{l} L\left(\left\{u_{k}\right\},\left\{l_{k}\right\},O\right)=\alpha {\sum_{k=1}^{K}\left.\left\|\frac{\partial \left(\delta \left(t\right)+\frac{j}{\pi t}\right)*u_{k}\left(t\right)}{\partial t}\exp\left(-jl_{k}t\right)\right.\right\|}_{2}^{2}+\\ {\left\|f\left(t\right)-\sum_{k=1}^{K}u_{k}\left(t\right)\right\|}_{2}^{2}+\langle O\left(t\right),f\left(t\right)-\sum_{k=1}^{K}u_{k}\left(t\right)\rangle \end{array}$$

where, *u_k_* is each IMF component; *l_k_* is the center frequency of each IMF component, Hz; *O* is the Lagrange multiplication operator; *f*(*t*) is the original signal; *δ*(*t*) is the Dirac function; *K* is the decomposition modulus; *α* is the penalty factor.

According to Equations (34)–(36), *O*, *u_k_*, and *l_k_* are iteratively updated until Equation (37) is satisfied, and *K* modal components are obtained:

(34) $${\hat{O}}^{n+1}\left(l\right)={\hat{O}}^{n}\left(l\right)+\tau \left(\hat{f}\left(l\right)-\sum_{k=1}^{K}{\hat{u}}_{k}^{n+1}\left(l\right)\right)$$

(35) $${\hat{u}}_{k}^{n+1}\left(l\right)=\frac{\hat{f}\left(l\right)-\sum_{i<k}{\hat{u}}_{i}^{n+1}\left(l\right)-\sum_{i>k}{\hat{u}}_{i}^{n}\left(l\right)+\frac{\hat{O}\left(\omega \right)}{2}}{1+2\alpha {\left(l-l_{k}^{n}\right)}^{2}}$$

(36) $$l_{k}^{n+1}=\frac{\int_{0}^{\infty }l{\left|{\hat{u}}_{k}^{n+1}\left(l\right)\right|}^{2}dl}{\int_{0}^{\infty }{\left|{\hat{u}}_{k}^{n+1}\left(l\right)\right|}^{2}dl}$$

(37) $$\sum_{k=1}^{K}\frac{{\left\|{\hat{u}}_{k}^{n+1}-{\hat{u}}_{k}^{n}\right\|}_{2}^{2}}{{\left\|{\hat{u}}_{k}^{n}\right\|}_{2}^{2}}<\varepsilon$$

In Formula (34) to (36), *n* is the number of iterations; ^ represents Fourier transform; *τ* is the noise tolerance coefficient; *ε* is the discrimination accuracy, which is usually set to 1 × 10^−6^.

Among the parameters that need to be set in the VMD algorithm, *K* and *α* have a greater impact on the decomposition effect, while *τ* and *ε* have a smaller impact. Therefore, it is necessary to find a suitable combination of *K* and *α* to achieve the optimal VMD decomposition effect.

### 3.2. Optimization of Penalty Factor α and Decomposition Modulus K

**Figure 5:**
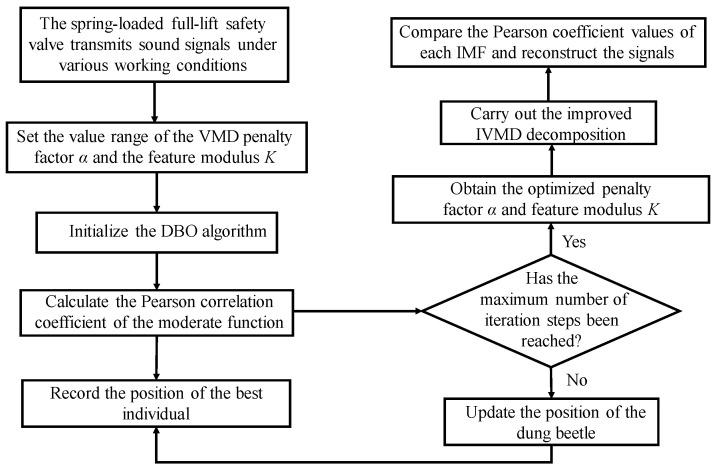
Flowchart of optimization of penalty factor *α* and decomposition modulus *K*.

For the optimization problem of the decomposition modulus *K* and penalty factor *α* of the VMD algorithm, the number of optimized parameters is the same as the optimization of the adjustable parameters of the IWTD function, and the purpose is to reduce the noise of the internal leakage acoustic emission signal of the spring full-open safety valve. Therefore, the IDBO algorithm is used to optimize the penalty factor *α* and decomposition modulus *K* of the VMD algorithm, and the process is shown in Figure 5.

## 4. IWTD-IVMD Composite Noise Reduction Overall Process

**Figure 6:**
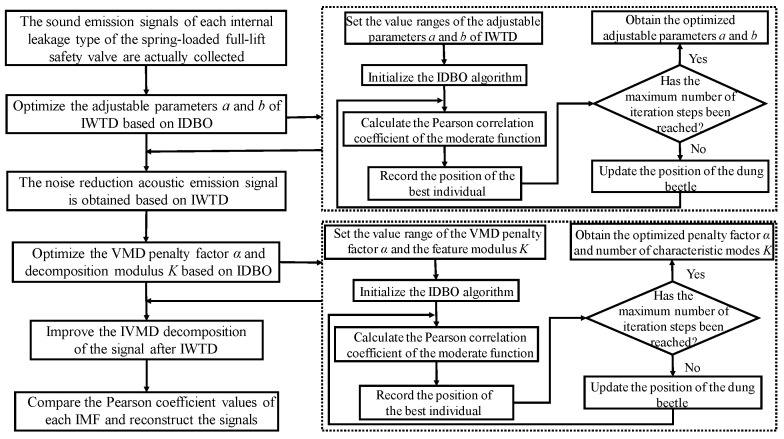
IWTD-IVMD noise reduction overall flow chart.

The overall process of the IWTD-IVMD composite denoising algorithm is shown in Figure 6. First, the actual acoustic emission signals of various types of internal leakage of the spring full-open safety valve are taken as input, and the adjustable parameters *a* and *b* of the IWTD function are optimized based on the IDBO algorithm. The acoustic emission signals after preliminary denoising are obtained based on the IWTD algorithm after parameter optimization. Taking the internal leakage acoustic emission signals after preliminary denoising by the IWTD algorithm as input, the penalty factor *α* and decomposition modulus *K* of VMD decomposition are optimized based on the IDBO algorithm. The acoustic emission signals are characteristically decomposed based on the IVMD algorithm after parameter optimization, and the Pearson coefficient values of each IMF are compared. The IMF with a larger Pearson coefficient is selected for signal reconstruction to obtain the final denoised internal leakage acoustic emission signals of the spring full-open safety valve.

## 5. Data Sources and Data Preprocessing

### 5.1. Research Objects and Operating Parameters

**Figure 7:**
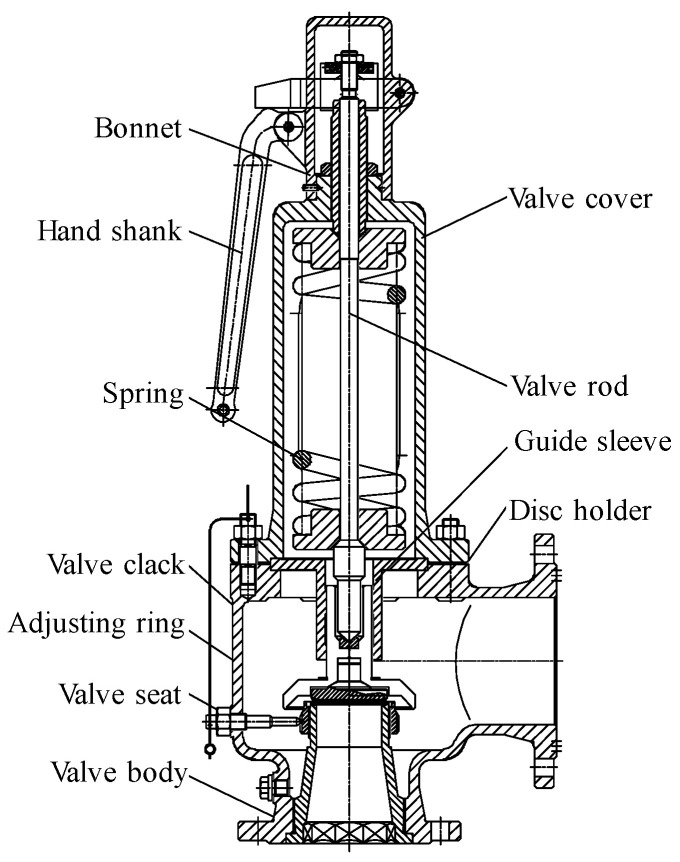
DN100 PN25 spring full-lift safety valve 2D drawing.

The typical model DN100 PN25 spring full-lift safety valve is selected as the research object, and the two-dimensional diagram is shown in Figure 7. The spring full-lift safety valve is mainly composed of valve body, valve cover, valve stem, spring, valve core, valve seat, and other components. The set pressure is 2.5 MPa, the discharge pressure is less than or equal to 2.575 MPa, and the return seat pressure is greater than or equal to 2.25 MPa. The valve seat and valve core sealing surface of the spring full-lift safety valve are manually polished, the surface roughness reaches *Ra* < 0.4 μm, and the appearance presents a mirror effect. Under the action of the spring force, the valve core and the valve seat sealing surface reach a sealing pressure ratio, and the safety valve is in a closed state at this time. When the medium pressure before the valve increases and approaches the set pressure of the safety valve, the valve core pre-opens and leaks forward. When the medium pressure reaches the starting pressure, the medium force on the safety valve core is greater than the spring force, and the safety valve opens rapidly until it reaches full opening. At this time, the safety valve fully opens to release the medium pressure. After a certain period of time, the medium pressure in front of the valve gradually decreases, the spring force overcomes the medium pressure, and the valve core returns to its seat and closes again.

### 5.2. Experimental Study on Different Types of Internal Leakage of Safety Valves

#### 5.2.1. Damage Types of Different Valve Seat Sealing Surfaces

**Figure 8:**
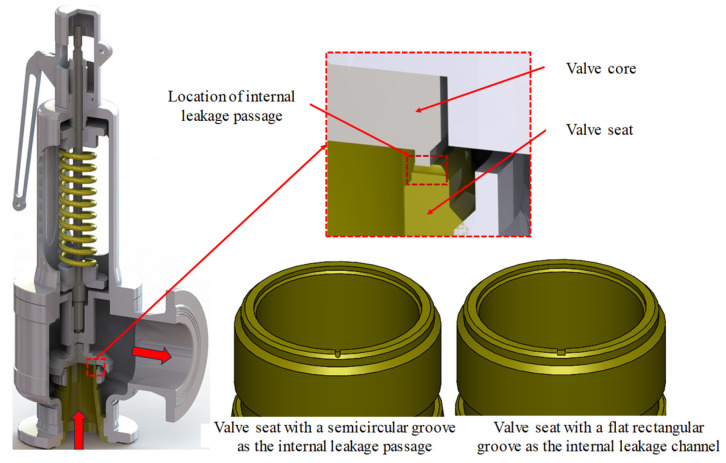
DN100 PN25 spring full-lift safety valve internal leakage three-dimensional model.

According to a large number of the actual inspection cases by the safety valve inspection department of the Guangdong Special Equipment Inspection Institute, a cooperative unit, the internal leakage of the spring full-open safety valve is mostly formed by erosion or impact of the valve seat sealing surface, which is approximately a semicircular internal leakage channel or a flat square internal leakage channel formed by long-term wear, and there are various forms such as a single internal leakage groove and multiple internal leakage grooves. The semicircular and flat square internal leakage grooves are shown in Figure 8. Through precise measurement, the equivalent cross-sectional area of the damaged internal leakage groove of the measured valve seat sealing surface is mostly between the semicircular area of *Φ*1 mm~*Φ*2 mm, and the length-to-width ratio of the approximate flat square groove is about 6:1. Different forms of internal leakage grooves on the valve seat sealing surface were processed, respectively, and the parameters are shown in Table 1. Single semicircular internal leakage groove valve seats with diameters of *Φ*1 mm and *Φ*2 mm and single flat square internal leakage groove valve seats with cross sections of 1.53 × 0.26 mm and 3.07 × 0.51 mm were processed, respectively.

#### 5.2.2. Safety Valve Internal Leakage Test of Different Types

**Figure 9:**
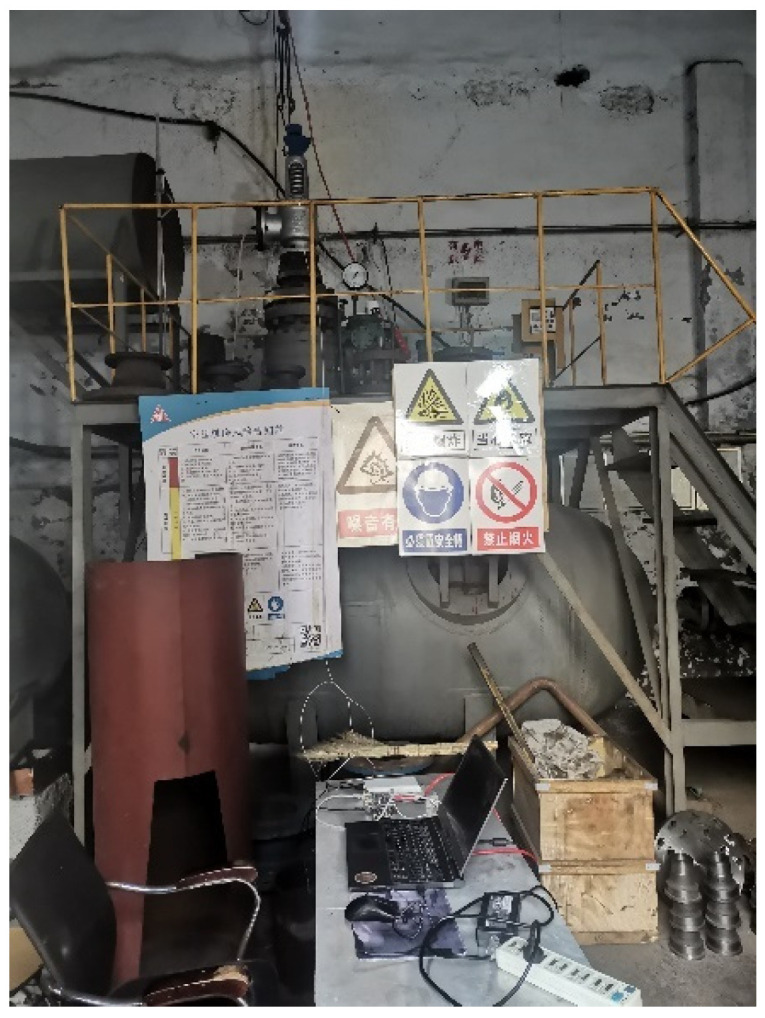
Safety valve internal leakage test site photo.

Therefore, for the internal leakage test of the PN25 DN100 spring full-lift safety valve, the air pressure before the valve is controlled at 2.25 MPa. The test site is shown in Figure 9. The compressor provides the pressure gas source, which reaches the pressure storage tank through the control valve and the bypass valve. When the spring full-lift safety valve leaks, the compressed gas flows to the safety valve through the pressure storage tank, and flows to the downstream of the system through the leakage groove of the safety valve seat to generate an acoustic emission signal. The spring full-lift safety valves equipped with valve seats of different internal leakage types are installed on the test bench in sequence. The acoustic emission sensor is arranged on the upper end face of the flange of the spring full-lift safety valve, and the developed small FPGA high-frequency acoustic emission acquisition system is used to collect high-frequency acoustic emission signals of different internal leakage types of the spring full-lift safety valve. The internal leakage flow of the spring full-lift safety valve is led out with a hose, and the internal leakage rate of the safety valve is measured using a high-precision rotor flowmeter with a small measurement range.

**Figure 10:**
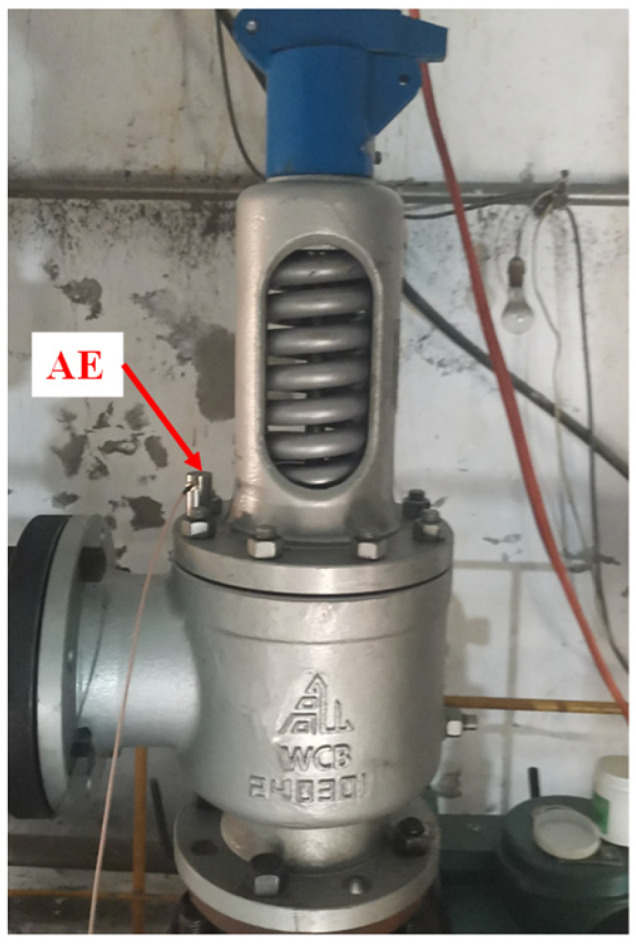
Schematic diagram of acoustic emission acquisition sensor location.

**Figure 11:**
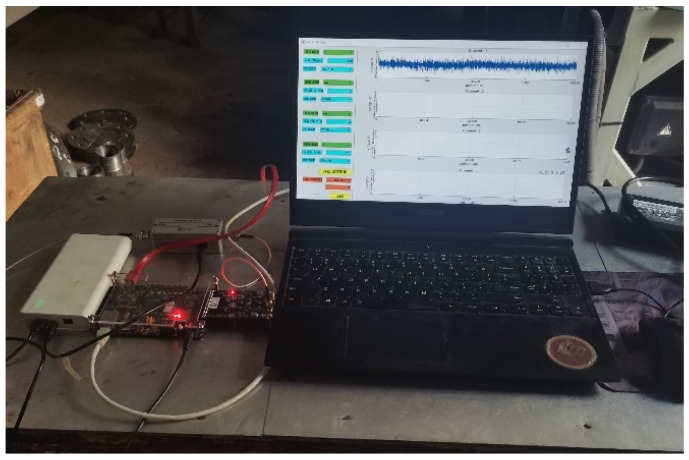
Schematic diagram of acoustic emission acquisition system.

The actual picture of the acoustic emission sensor measuring point on the flange end face of the spring full-lift safety valve is shown in Figure 10, and the FPGA high-frequency acoustic emission acquisition system and the host computer on-site acquisition interface are shown in Figure 11. In order to ensure that the acoustic emission signal is not distorted, the acquisition frequency should be set to more than five times the actual frequency of the measured signal, so the acquisition frequency of 500 kHz is selected.

Since the actual acoustic emission signal cannot separate the pure components, the effectiveness of the method is verified by constructing synthetic data of the known signal + controllable noise. The simulated signal is based on the time–frequency characteristics of the measured leakage pulse, generating a transient waveform with adjustable amplitude/frequency, and injecting noise by mixing Gaussian noise (sensor noise) and 1/f noise (mechanical vibration) in proportion. Under the condition of known real signal, the following is calculated: theoretical SNR = 10log10 (signal energy/noise energy); characteristic fidelity = number of retained leakage pulses/total number of injected pulses, which is used as an evaluation index.

The measured values of the internal leakage rate of different internal leakage types of the spring full-open safety valve are shown in Table 2. For the spring full-open safety valve with a single internal leakage groove, under the working condition of 2.25 MPa before the valve, the internal leakage rate of the semicircular groove with a diameter of *Φ*2 mm is the largest, reaching 0.00209 kg/s. The internal leakage rate of the single square groove with a diameter of 1.53 × 0.26 mm is the smallest, with a value of 0.00035 kg/s. The internal leakage rate of the single square groove with a cross-sectional area of 3.07 × 0.51 mm, which is the same as the semicircular groove with a diameter of *Φ*2 mm, is 0.00178 kg/s, and the internal leakage rate is lower than the internal leakage rate of the semicircular groove with the same cross-sectional area. The internal leakage rate of the single square groove with a cross-sectional area of 1.53 × 0.26 mm is also lower than the internal leakage rate of the single semicircular groove with the same cross-sectional area of *Φ*1 mm. Therefore, it can be concluded that for the internal leakage groove with the same cross-sectional area, the flow resistance of the square internal leakage groove is greater than that of the semicircular internal leakage groove. Under the same pressure before the valve, the internal leakage rate of the square internal leakage groove of the valve seat with the same cross-sectional area is smaller than the internal leakage rate of the semicircular internal leakage groove.

### 5.3. Wavelet Time–Frequency Analysis of Acoustic Emission Signals of Different Endoleak Types

For the collected safety valve non-internal leakage acoustic emission time domain signals and various internal leakage types of acoustic emission time domain signals, each signal is randomly intercepted for a 0.1 s time period, and wavelet time–frequency transformation is performed to obtain a two-dimensional wavelet time–frequency spectrum of each signal for 0.1 s time period. Preliminary synthetic tests show that when the input is SNR = 10 dB, the method can recover 92% of the leakage pulses, and the SNR after noise suppression is increased to 18.3 dB, which is basically consistent with the trend of measured data. However, synthetic verification can only reflect the potential of the method under ideal conditions, and the actual performance is affected by the characteristics of the sensor and the installation conditions.

**Figure 12:**
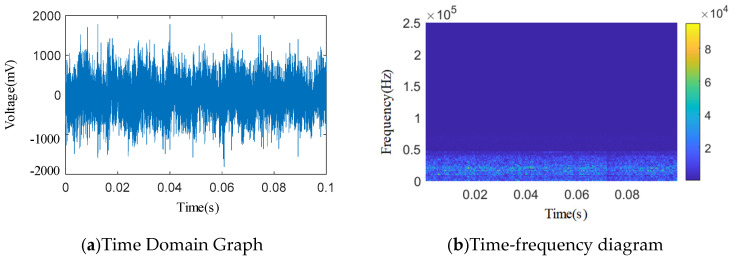
Leakage-free time domain and wavelet time–frequency diagram.

**Figure 13:**
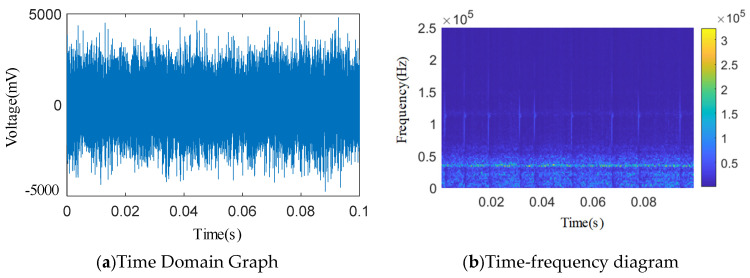
*Φ*2 mm single semicircular groove time domain and wavelet time–frequency diagram.

**Figure 14:**
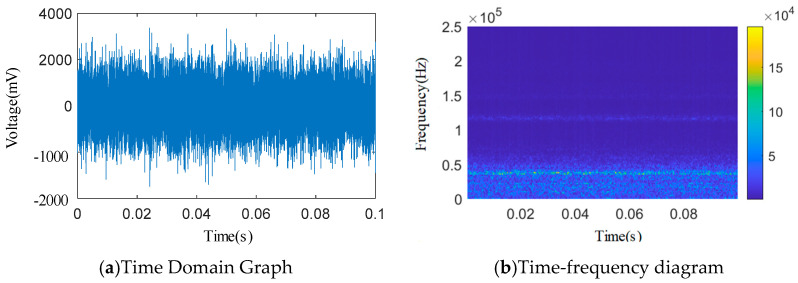
*Φ*1 mm single semicircular groove time domain and wavelet time–frequency diagram.

**Figure 15:**
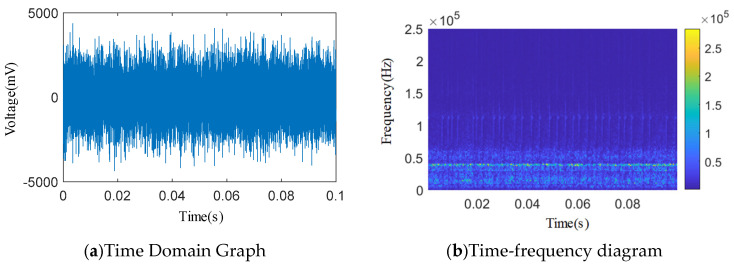
Time domain and wavelet time–frequency diagram of a 3.07 × 0.51 mm single square slot.

**Figure 16:**
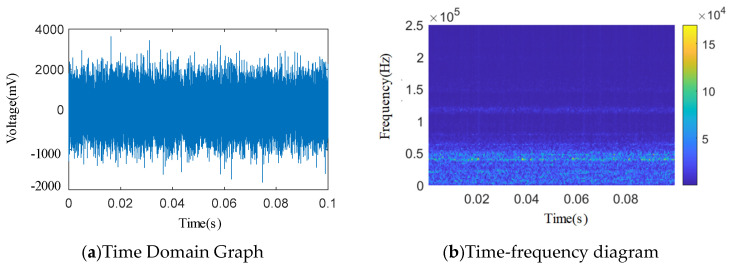
1.53 × 0.26 mm single square slot time domain and wavelet time–frequency diagram.

As shown in Figure 12, Figure 13, Figure 14, Figure 15 and Figure 16, from the wavelet time–frequency spectrum, we can see that: There is a significant overlap in the frequency distribution of the safety valve internal leakage acoustic emission signal and the ambient noise. Although most of the energy of the acoustic emission signal without internal leakage is concentrated in the relatively low-frequency band, there is also a lot of energy distribution in the ultrasonic frequency band above 20 kHz. Although the high-energy area of the acoustic emission signal of different internal leakage types is concentrated in the ultrasonic frequency band of 20 kHz~50 kHz, there is also a lot of energy distribution below the ultrasonic frequency band of 20 kHz. This overlapping characteristic makes it difficult for filtering methods that rely solely on frequency thresholds to effectively separate signals from noise. Therefore, a more sophisticated time–frequency domain joint-processing strategy is needed. This paper adopts two methods: IWTD’s adaptive threshold adjustment and IVMD’s modal component screening to distinguish the overlap of signals in the frequency band.

(1) Adaptive threshold adjustment of IWTD

The improved wavelet threshold function (IWTD) dynamically adjusts the threshold processing method through dual parameters (*a*, *b*). For overlapping frequency bands below 20 kHz, when the wavelet coefficients are in the fuzzy area between the signal and the noise, containing both leakage signal characteristics and noise, IWTD does not directly set it to zero. This is different from the hard threshold. Instead, it uses an exponential function to smoothly attenuate the noise component and retain the weak internal leakage signal characteristics. For high frequency bands above 20 kHz, IWTD approaches the hard threshold through asymptotic characteristics (when *ω* → ∞, $\hat{\omega }$ → *ω*), retaining the strong signal component to the maximum extent.

(38) $$\hat{\omega }=\left\{\begin{array}{l} \mathrm{sign}(\omega )\left[\left|\omega \right|+2\lambda \frac{\left(a-1\right)}{e^{(1-b){\left(\omega -\lambda \right)}^{2}}+1}\right],\;\left|\omega \right|\geq \lambda \;\\ \mathrm{sign}(\omega )a\left|\omega \right|e^{(1-b){\left(\omega -\lambda \right)}^{2}},\;\left|\omega \right|<\lambda \end{array}\right.$$

(2) Modal component screening of IVMD

After preliminary noise reduction by IWTD, IVMD decomposes the signal into IMF components through optimized *K* and *α*. Even if the frequency bands overlap, the IMF component (IMF1) of the internal leakage signal shows a more stable energy concentration in the time–frequency domain (such as a continuous peak of 30~40 kHz), while the noise component (IMF2, IMF3) has dispersed energy and decays over time; by screening the Pearson coefficient (only retaining IMF1 with R > 0.9), the noise-dominant component in the overlapping frequency band can be further eliminated to achieve signal purification.

Although this method can effectively deal with the problem of frequency band overlap, in extreme noise scenarios (such as 10~20 kHz noise energy accounted for >50%), the signal feature retention rate will drop below 85%. In the future, deep learning will be combined to further improve the feature differentiation ability of overlapping frequency bands.

The energy distribution between different types of endoleaks is seriously stacked, and there is no clear feature to distinguish them. The two-dimensional wavelet time–frequency diagrams of acoustic emission signals without endoleaks and different types of endoleaks have problems such as a poor image recognition effect and a lack of prominent features. Therefore, it is necessary to study signal denoising and feature extraction.

## 6. Analysis of Noise Reduction Results of Safety Valve Internal Leakage Signal

### 6.1. Feasibility Analysis of Noise Reduction Process

(1) Hardware configuration and signal pre-processing

The hardware platform used is Intel(R) Core(TM) i5-10200H CPU @ 2.40 GHz 2.40 GHz, 16 GB DDR4 memory, Windows 10 system, and the internal leakage signal is analyzed. A total of 20 segments of 0.1 s signals are randomly selected for each type, and the sampling frequency is 500 KHz. The total processing time of each segment of signal is determined, including IWTD parameter optimization, IVMD parameter optimization, and noise reduction calculation.

After experiments, the average processing time of each 0.1 s signal is 316 ms, which is lower than the time of industrial offline detection, indicating the feasibility of this method. The IDBO optimization process accounts for 68% of the total time (of which IWTD parameter optimization accounts for 32% and IVMD parameter optimization accounts for 36%), and noise reduction calculation accounts for 32%. As the amount of data increases, the total time consumption increases approximately linearly, and the average time consumption for each 20 segments is 6.3–6.5 s, which can meet the requirements of batch data processing.

Acoustic emission signals of spring full-lift safety valves and safety valves without internal leakage with different forms of valve seat sealing surface damage were collected to obtain various types of acoustic emission time domain signals. Wavelet transform was performed on the acoustic emission time domain signals to obtain wavelet two-dimensional time–frequency spectrum of acoustic emission signals of different internal leakage types. By analyzing the wavelet two-dimensional time–frequency spectrum, the main frequency distribution range of the acoustic emission signal energy of the spring full-lift safety valve without internal leakage and the different internal leakage types was preliminarily obtained. The wavelet time–frequency spectrum of no internal leakage and various internal leakage types both show wide-band characteristics. The energy distribution frequency band of the acoustic emission signal without internal leakage is lower than the energy distribution frequency band of the acoustic emission signal of various internal leakage types as a whole, but there are still a lot of overlapping areas between the two at around 20 kHz. It can be seen from the wavelet two-dimensional time–frequency spectrum that the energy of the acoustic emission signal of each internal leakage type has a lot of overlapping areas in the frequency domain direction, and there is no obvious feature difference in the two-dimensional time–frequency spectrum. Affected by the compressor vibration noise and the pipeline vibration noise in the acoustic emission signal collection environment of the spring full-lift safety valve, the acoustic emission signals of various internal leakage types still have a high energy distribution in the relatively low frequency band less than 20 kHz.

Taking a 0.1 s signal in the time domain signal of the leakage sound emission of a *Φ*1 mm single semicircular groove of a spring fully-open safety valve as an example, the IWTD-IVMD composite noise reduction processing method is gradually demonstrated.

(2) IDBO optimization verification of IWTD-IVMD

To ensure the advantages of the IDBO optimization algorithm in IWTD-IVMD optimization, the IDBO optimization algorithm is compared with standard DBO, particle swarm optimization algorithm (PSO), and genetic algorithm (GA) before denoising. The leakage signal of *Φ*1 mm single semicircular groove is used as a sample to optimize the parameters *a*, *b*, *K*, *α* of IWTD-IVMD. The optimization target is the maximum Pearson coefficient, the uniform population size is 25, the maximum number of iterations is 50, and the search range is *a*, *b* ∈ [0, 1], *K* ∈ [2, 32] and *α* ∈ [10, 5000]. The optimal Pearson coefficient, the number of convergence iterations, and the average time (10 control tests) are used as evaluation indicators. The experimental results are shown in Table 3.

The results show that the optimal Pearson coefficient of the composite denoising effect after IDBO optimization is 0.912, which is significantly higher than GA, PSO, and standard DBO, indicating that it effectively retains the main features of the internal leakage signal through more accurate parameter optimization (*a*, *b* of IWTD and *K*, *α* of IVMD), and verifies the practical value of the three improvement strategies (greedy lens imaging reverse learning, PID perturbation, and curve adaptive weight) in improving the performance of composite denoising. In terms of the number of iterations, IDBO has the least number of iterations in IWTD and IVMD parameter optimization (39 and 17 steps respectively), which is 27%~47% less than the other three classic optimization algorithms, indicating that it converges faster and can find the global optimal parameters in fewer iterations; the total time consumption is lower than other algorithms, especially 25% less than GA, thanks to the optimization of the search path by the improved algorithm, which reduces invalid iterations. After experimental verification, the three improvement strategies effectively improved the improved DBO, verifying the advantages of IDBO compared with traditional optimization algorithms. It not only performs better in the single parameter optimization stage but also improves the performance of the entire composite denoising process through accurate optimization.

### 6.2. Analysis of IWTD Noise Reduction Results

A 0.1 s signal in the time domain signal of the sound leakage emission in a *Φ*1 mm single semicircular slot is used as the input of the IDBO optimization algorithm, and the Pearson correlation coefficient is used as the fitness function to obtain the optimal values of the IWTD adjustable parameters a and b corresponding to the maximum Pearson coefficient in a given iteration step. First, the relevant parameter values need to be configured. The value range of the adjustment parameter a is given as [0, 1], the value range of the adjustment parameter b is also given as [0, 1], and the number of optimization variables is 2. According to the literature on parameter optimization of the classic DBO algorithm, the maximum number of iterations for the preliminary optimization calculation is usually 50~100, and the number of dung beetle populations is 20~30. If the fitness function curve cannot converge within this range of iteration steps, the number of iteration steps will continue to be expanded. Considering the computational efficiency, the number of iterations of the improved DBO algorithm is set to 50, and the number of dung beetle populations is set to 25. All relevant parameter configurations are shown in Table 4.

**Figure 17:**
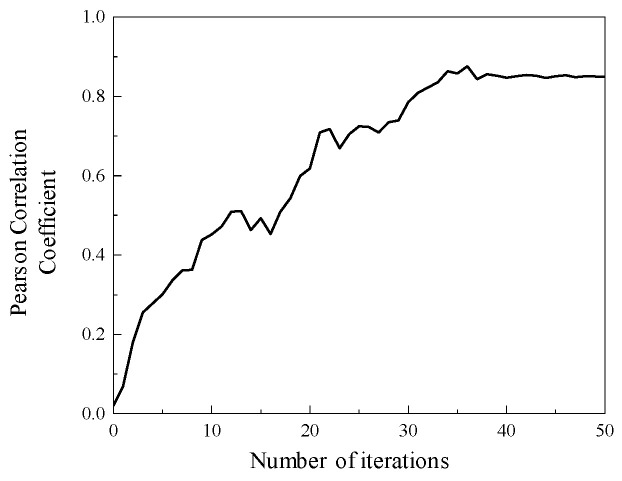
IDBO algorithm optimization process.

The optimization process of the IDBO algorithm for the adjustable parameters *a* and *b* of the IWTD function is shown in Figure 17. When the number of calculation iterations is 39, the Pearson coefficient of the fitness function tends to be stable, and the Pearson coefficient value is 0.873. When the maximum number of iterations is calculated, the optimization results of the IWTD adjustable parameters *a* and *b* are obtained, which are as follows: adjustable parameters *a* = 0.23, *b* = 0.79.

**Figure 18:**
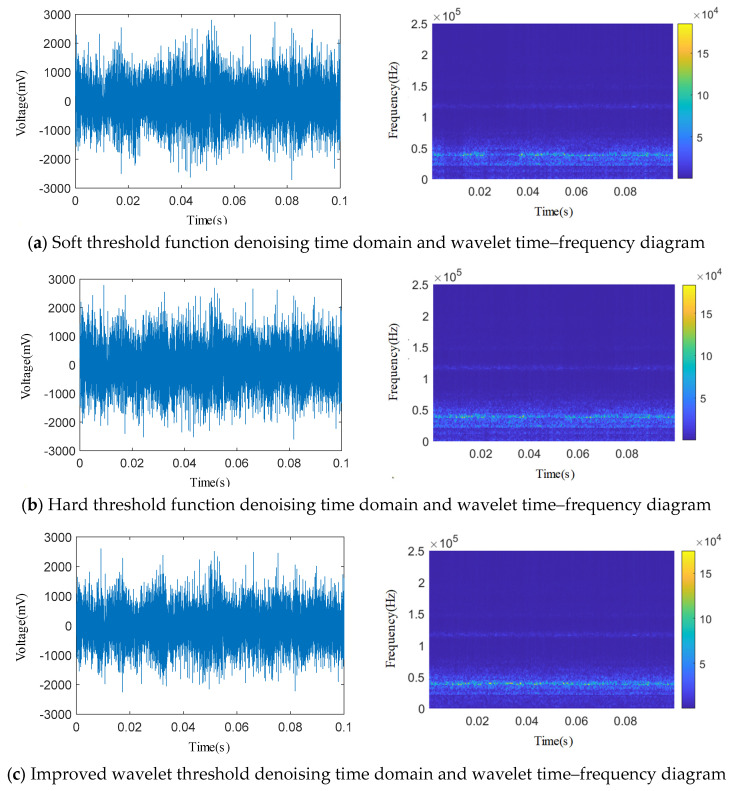
Comparison of denoising effects of three wavelet threshold functions.

The adjustable parameters *a* = 0.23 and *b* = 0.79 in the IWTD function are set to perform IWTD function denoising on a certain 0.1 s length signal in the *Φ*1 mm single semicircular groove internal leakage acoustic emission time domain signal of the spring full-open safety valve, and the classic soft and hard threshold functions are used to denoise this signal at the same time. The acoustic emission time domain signal reconstructed by three different threshold functions is subjected to wavelet time–frequency transformation, and the time domain signal and wavelet two-dimensional time–frequency diagram are shown in Figure 18. As shown in Figure 18, after denoising by soft threshold and hard threshold functions, the mechanical noise signals such as compressor vibration, pipeline vibration and other pipe system throttling element vibration with low energy in the 0~20 kHz frequency band of the acoustic emission signal are partially removed. However, in the 20~60 kHz frequency band, some high-energy internal leakage frequency band signals are also removed, and the overall denoising effect is not good. After IWTD denoising, the 20~60 kHz internal leakage frequency band with large energy in the acoustic emission signal is well retained, and most of the low-frequency mechanical vibration noise is removed, and the denoising effect is good.

The Pearson coefficient and other indicators of the reconstructed signal after denoising using three different threshold functions were calculated and compared, and the results are shown in Table 5. It can be seen from Table 4 that the Pearson coefficient of the acoustic emission signal reconstructed after denoising with the soft threshold function is 0.752; the Pearson coefficient of the acoustic emission signal reconstructed after denoising with the hard threshold function is 0.791; the Pearson coefficient of the acoustic emission signal reconstructed after denoising with the IWTD function is 0.853. Therefore, combined with the Pearson coefficient evaluation index and the wavelet two-dimensional time–frequency diagram analysis, and compared with the classic soft and hard threshold functions, the IWTD function optimized by the IDBO algorithm can better retain the main features of the original signal, has a larger Pearson coefficient, and has a better denoising effect.

### 6.3. Analysis of IVMD Feature Decomposition Effect

The time domain signal of the sound leakage emission of the *Φ*1 mm single semicircular groove of the spring full-open safety valve after denoising by the IWTD function is used as the input of the IDBO optimization algorithm, and the Pearson correlation coefficient is used as the fitness function to obtain the optimal value of the VMD decomposition penalty factor *α* and the decomposition modulus *K* corresponding to the maximum Pearson coefficient in a given iteration step. First, it is necessary to configure the values of relevant parameters. According to the relevant literature at home and abroad, the value range of the penalty factor *α* is usually [10, 5000], the value range of the decomposition modulus *K* is usually [2, 32], and the number of optimization variables is 2. The same as the IWTD function parameter optimization, considering the computational efficiency, the number of iteration steps of the IDBO algorithm is set to 50, and the number of dung beetle populations is set to 25. If the fitness function curve cannot converge within this range of iteration steps, continue to expand the number of iteration steps. All relevant parameter configurations are shown in Table 6.

**Figure 19:**
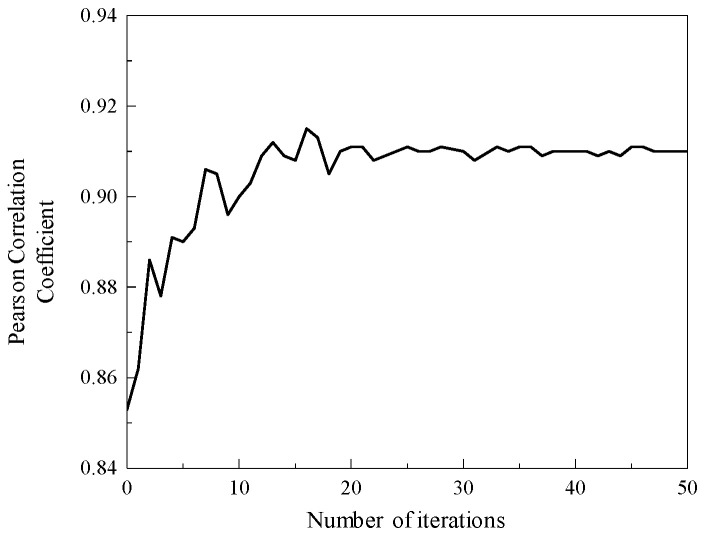
IDBO algorithm optimization process.

The optimization process of the IDBO algorithm for the VMD decomposition penalty factor *α* and the decomposition modulus *K* is shown in Figure 19. When the number of calculation iterations is 17, the Pearson coefficient of the fitness function tends to be stable, and the value is 0.912. After the maximum number of iterations is calculated, the optimization results of the VMD decomposition penalty factor *α* and the decomposition modulus *K* are obtained, which are as follows: adjustable parameters *α* = 3000, *K* = 3. Therefore, the IVMD decomposition algorithm is obtained by optimizing the parameters of the IDBO algorithm.

**Figure 20:**
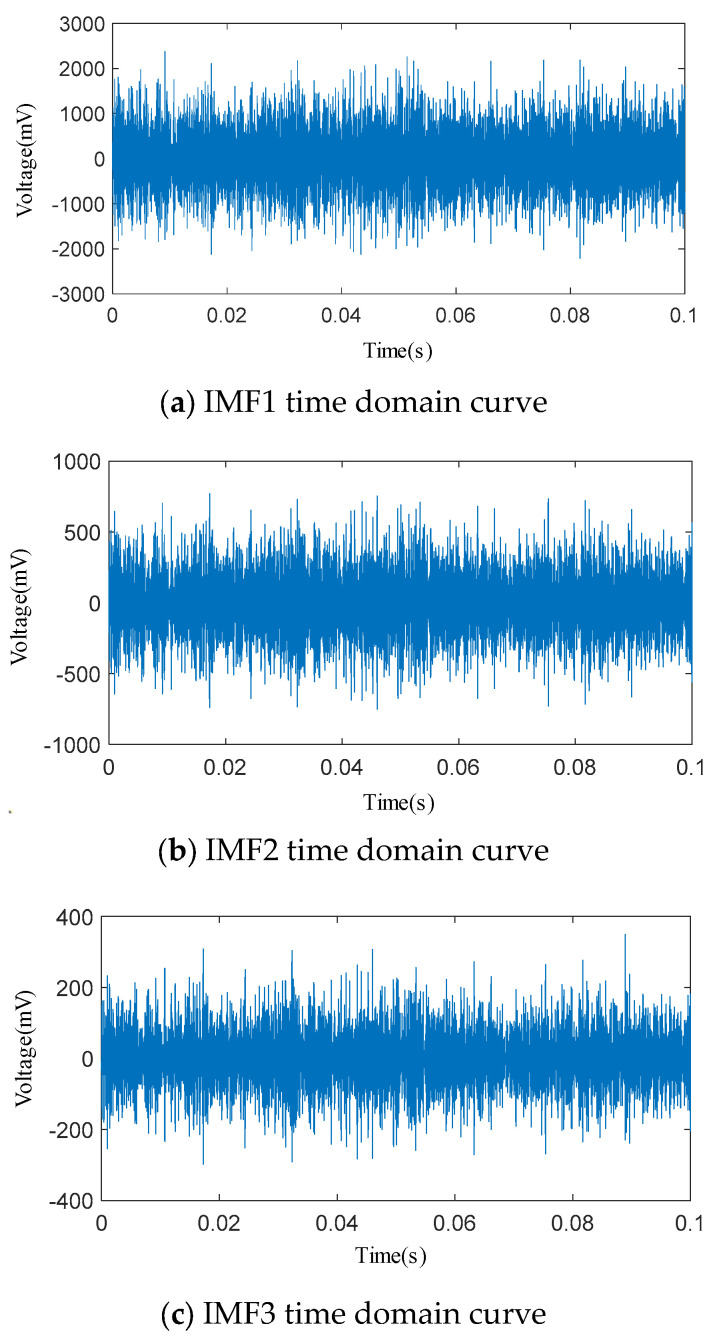
Time domain curves of various IMFs after IVMD decomposition.

**Figure 21:**
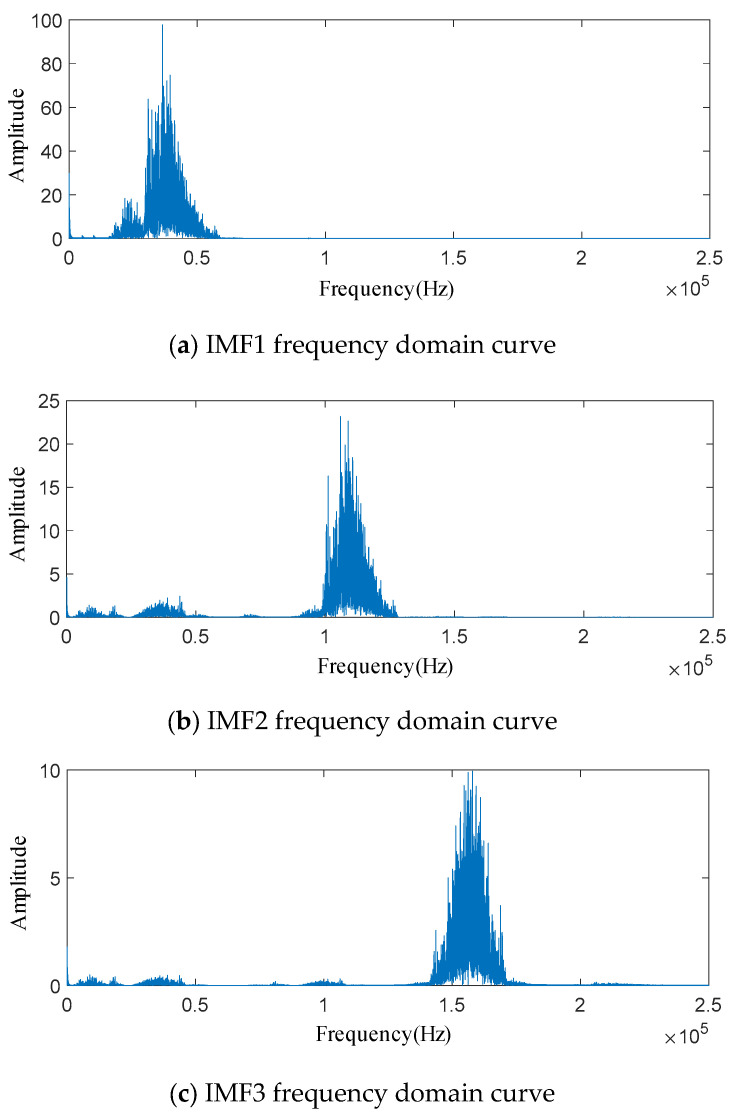
Frequency domain curves of each IMF after IVMD decomposition.

**Figure 22:**
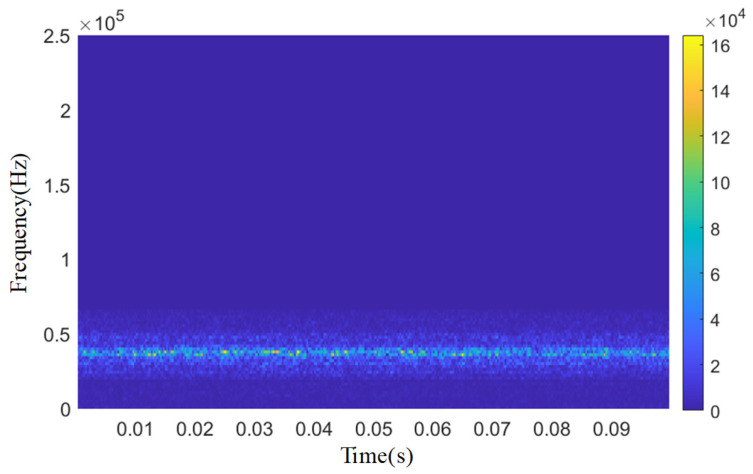
Time–frequency diagram of the *Φ*1 mm single semicircular internal leakage groove after final noise reduction.

The penalty factor *α* is set to 3000 and the decomposition modulus *K* is set to 3. The time domain signal of the acoustic emission of the spring full-open safety valve *Φ*1 mm single semicircular groove internal leakage condition after IWTD denoising is decomposed by IVMD to obtain the time domain signals of different IMF components, as shown in Figure 20.The time domain signals of different IMF components are subjected to fast Fourier transform to obtain the spectrum diagram of different IMF components, as shown in Figure 21. It can be seen that the amplitude of the IMF1 spectrum curve is the largest, and the peak is distributed in the ultrasonic frequency band of 30~40 kHz. The peaks of the IMF2 and IMF3 spectrum curves are distributed in the ultrasonic frequency bands of 110~120 kHz and 150~160 kHz, respectively. The Pearson coefficients of the IMF1, IMF2, and IMF3 time domain curves are solved, respectively, as shown in Table 6. The Pearson coefficients of IMF1, IMF2, and IMF3 are 0.912, 0.613 and 0.452, respectively, and the Pearson coefficient of IMF1 is much larger than that of IMF2 and IMF3. Therefore, the IMF1 time domain signal is selected as the final denoised and reconstructed signal, and wavelet time–frequency transform is performed, as shown in Figure 22. Analysis of the wavelet time–frequency diagram shows that after IVMD decomposition, the energy highlight area in the acoustic emission signal is concentrated in the ultrasonic frequency band of 30~40 kHz, and the wavelet two-dimensional time–frequency diagram has obvious characteristics.

According to the comparison of characteristic indicators of each IMF component in Table 7, a high Pearson coefficient (>0.9) appears simultaneously with high SNR, low RMSE, and low spectrum flatness, indicating that the characteristics of the inner leakage signal are retained rather than noise (such as the 30–40 kHz energy concentration of IMF1); the Pearson coefficient combined with indicators such as SNR and RMSE can effectively avoid the risk of retaining noise to maintain correlation and comprehensively evaluate the noise reduction performance.

To further illustrate the advantages of IWTD-IVMD, based on the comparison of the original soft and hard threshold functions, the improved modal decomposition method (CEEMDAN) and the standard VMD technology are compared.

Fully adaptive noise ensemble empirical mode decomposition (CEEMDAN): By introducing adaptive white noise and multiple integrations (the number of integrations is set to 100 times in the experiment), the modal aliasing problem of traditional EMD is effectively alleviated. The threshold processing stage uses the noise standard deviation of 0.2 as the benchmark, and the separation of noise and signal is achieved by dynamically adjusting the threshold strength. This method is particularly suitable for acoustic emission signals with non-stationary characteristics, but its computational cost increases significantly with the increase in the number of integrations.

Unoptimized standard VMD: The fixed parameter modal number *K* = 5 and the penalty factor *α* = 2000 are used for signal decomposition. Although the computational overhead of parameter optimization is avoided, the adaptability to complex acoustic emission signals is poor. Experiments show that the IMF components decomposed often have frequency band overlap, resulting in incomplete leakage feature extraction. After experimental comparison, as shown in Table 8, the Pearson coefficient of IWTD-IVMD is much higher than that of the other three optimization algorithms. The SNR trend is consistent with the Pearson coefficient, which is better than several other optimization algorithms. The results confirm the key impact of parameter optimization on VMD performance.

### 6.4. IWTD-IVMD Noise Reduction Results of All Internal Leakage-Type Acoustic Emission Signals

The optimal parameters of different leakage types show certain commonalities: the values of *a* are concentrated in 0.21~0.25, *b* is concentrated in 0.78~0.82, *K* is mainly 3 or 4, and *α* is concentrated in 2800~3200. This shows that despite the non-stationarity of the signal, the parameter adaptation range of the same leakage scenario is stable. Further analysis shows that the parameter difference is mainly due to the leakage channel morphology: the square slot has a larger flow resistance, and the energy distribution of the internal leakage acoustic emission signal is more concentrated, so the K value is slightly higher to enhance the high-frequency component decomposition ability; and α increases with the increase in leakage, which is consistent with the need for a higher penalty factor for high-energy signals to suppress modal aliasing.

**Figure 23:**
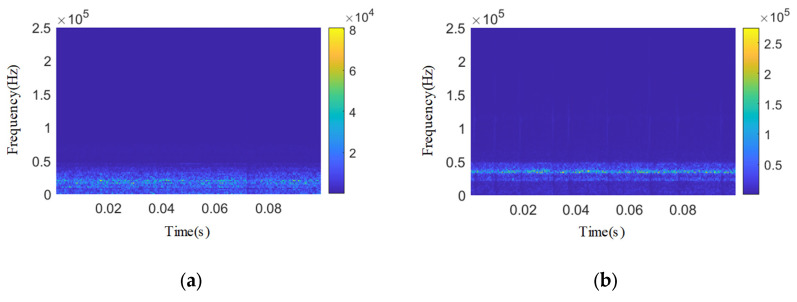

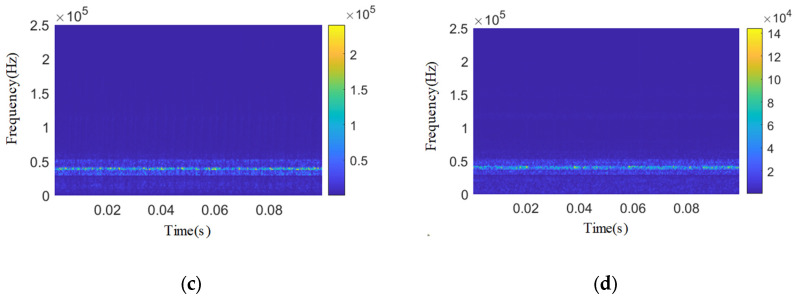
Wavelet two-dimensional time–frequency diagram of other types of acoustic emission signals after composite denoising. (**a**) No internal leakage. (**b**) *Φ*2 mm single semicircular internal leakage groove. (**c**) 3.07 × 0.51 mm single square inner leakage groove. (**d**) 1.53 × 0.26 mm single square inner leakage groove.

Based on the above rules, the default parameter combination is as follows: *a* = 0.23, *b* = 0.80, *K* = 3, *α* = 3000. The Pearson coefficient deviation of this combination is <0.02 in all leakage types, meeting the requirements of accuracy and efficiency. The optimal parameters for different leakage types are shown in Table 9:

The IWTD-IVMD composite noise reduction method is used to reduce the noise of a 0.1 s length time domain signal of several types of internal leakage other than the spring full-open safety valve without internal leakage and the *Φ*1 mm single semicircular internal leakage groove, and the time domain signals after composite noise reduction and reconstruction of each type of acoustic emission signal are, respectively, subjected to wavelet time–frequency transformation, as shown in Figure 23. The Pearson coefficient values of each stage of the composite noise reduction and reconstruction signal of each type of internal leakage are shown in Table 10. The acoustic emission signal in the no-internal-leakage state is essentially a background noise reference signal, including the following: stable vibration noise of the pipeline system (10~20 kHz), valve body micro-vibration in the closed state of the safety valve (20~30 kHz), environmental electromagnetic interference, etc. Its noise reduction goal is not to eliminate all noise, but to retain the stable time–frequency characteristics of the background noise to provide a reference for the identification of the internal leakage signal. Therefore, the noise reduction requirements for the no-internal-leakage signal are as follows: eliminate sudden interference and retain the stable background noise characteristics.

After IWTD-IVMD composite denoising and reconstruction, the Pearson coefficients of all endoleak types of acoustic emission signals are greater than 0.9 and close to 1. After denoising, the wavelet time–frequency graphs of each endoleak type of acoustic emission signals have obvious frequency band characteristics, showing obvious narrowband characteristics.

## 7. Conclusions

There are a lot of energy overlapping areas in the wavelet time–frequency spectrum of the original measured acoustic emission signal of each type of internal leakage of the spring full-open safety valve. The overall signal presents broadband characteristics, high noise content, and no obvious time–frequency characteristics. The existing wavelet soft and hard threshold function denoising methods have problems such as constant deviation outside the threshold, discontinuity at the threshold, and Gibbs effect during reconstruction. In view of the above problems, a composite denoising method combining IWTD-improved wavelet threshold function and IVMD decomposition algorithm is proposed.

In terms of improving the wavelet threshold function, an IWTD function with dual adjustable factors is proposed. This function is continuous, progressive, bias-free, and adjustable, overcoming the shortcomings of traditional soft and hard wavelet thresholds. Based on the improved dung beetle optimization algorithm, the maximum Pearson coefficient is taken as the optimization target, and the optimization is performed within the value range of the dual adjustable factors *a* and *b*, and the optimal value of the dual adjustable factors is obtained.

In terms of IVMD decomposition algorithm, taking the maximum Pearson coefficient as the optimization target, the improved dung beetle algorithm is used to optimize the traditional VMD algorithm parameters *K* and *α* within the value range, and the optimal values of *K* and *α* are obtained. The internal leakage acoustic emission signal after IWTD denoising is further decomposed based on the IVMD decomposition algorithm. The IMF component with a higher Pearson coefficient is reconstructed to obtain the internal leakage acoustic emission signal after composite denoising.

The Pearson coefficients of the acoustic emission signals of each type of internal leakage after IWTD-IVMD composite denoising are all greater than 0.9, which is much higher than traditional denoising methods such as soft and hard threshold functions. Therefore, the IWTD-IVMD composite denoising method can extract more main features of the measured spring full-open safety valve internal leakage acoustic emission signal, and has a good denoising effect.

## Figures and Tables

**Table 1:** Internal leakage forms of different valve seat sealing surfaces.

Working Conditions	Internal Leakage
Working conditions 1	The valve seat sealing surface is not damaged
Working conditions 2	*Φ*1 mm single semicircular groove
Working conditions 3	*Φ*2 mm single semicircular groove
Working conditions 4	1.53 × 0.26 mm single square slot
Working conditions 5	3.07 × 0.51 mm single square slot

**Table 2:** Inner leakage rate of different inner leakage types.

Internal Leakage Type	Internal Leakage Rate (kg/s)
*Φ*1 mm single semicircular groove	0.00043
*Φ*2 mm single semicircular groove	0.00209
1.53 × 0.26 mm single square slot	0.00035
3.07 × 0.51 mm single square slot	0.00178

**Table 3:** Comparison of different optimization algorithms.

Optimization Algorithm	Optimal Pearson Coefficient	IWTD Convergence Iteration Number	IVMD Iteration Number	Total Time (ms)
Standard DBO	0.869	45	32	638
PSO	0.853	40	38	693
GA	0.826	42	45	798
IDBO	0.912	39	17	587

**Table 4:** IDBO algorithm optimization parameter settings.

*a*	*b*	Optimizing the Number of Variables	Maximum Number of Iterations	Population Size
[0, 1]	[0, 1]	2	50	25

**Table 5:** Multi-index comparison of the noise reduction effects of three threshold functions.

Threshold Function	Pearson Coefficient	SNR (dB)	RMSE	Spectral Flatness
Soft threshold function	0.752	4.2	0.012	0.82
Hard threshold function	0.791	5.1	0.009	0.78
IWTD Function	0.853	7.5	0.005	0.65

**Table 6:** IDBO algorithm optimization parameter settings.

*α*	*K*	Optimizing the Number of Variables	Maximum Number of Iterations	Population Size
[10, 5000]	[2, 32]	2	50	25

**Table 7:** Comparison of characteristic indicators of each IMF component.

IMF Components	Pearson Coefficient	SNR (dB)	RMSE	Main Energy Frequency Band (KHz)
IMF1	0.912	12.3	0.003	30~40
IMF2	0.613	5.8	0.011	110~120
IMF3	0.452	3.2	0.018	150~160

**Table 8:** Multi-index comparison of different noise reduction methods.

Evaluation Indicators	Hard Threshold	CEEMDA	Standard VMD	IWTD-IVMD
Pearson coefficient	0.791	0.832	0.812	0.917
SNR (dB)	16.8	19.2	18.7	22.4
Calculation time (s)	0.12	3.45	2.87	5.87

**Table 9:** Comparison of optimal parameters for different leakage types.

Leak Type	*a*	*b*	*K*	*α*
No leakage	0.21	0.82	3	2800
*Φ*1 mm single semicircular groove	0.23	0.79	3	3000
*Φ*2 mm single semicircular groove	0.25	0.81	4	3200
3.07 × 0.51 mm single square slot	0.22	0.80	3	2900
1.53 × 0.26 mm single square slot	0.24	0.78	4	3100

**Table 10:** Pearson coefficient after various types of composite noise reduction.

Type	Pearson Coefficient of the Original Signal	Pearson Coefficient After IWTD Denoising	Pearson Coefficient After Composite Noise Reduction
No internal leakage	0.618	0.846	0.917
*Φ*1 mm single semicircular groove	0.625	0.853	0.912
*Φ*2 mm single semicircular groove	0.630	0.849	0.909
3.07 × 0.51 mm single square slot	0.622	0.850	0.911
1.53 × 0.26 mm single square slot	0.620	0.847	0.913

## Data Availability

The original contributions presented in this study have been included in the article. If you have any further questions, please contact the corresponding author.
